# Beyond population size: long-term changes in free-roaming cat population structure during TNR implementation in Seoul

**DOI:** 10.3389/fvets.2026.1887137

**Published:** 2026-09-23

**Authors:** Yoonju Cho, Jun Hyuk Byun, Bo Ram Hwang, Youngah Lee

**Affiliations:** 1 VIP Animal Medical Center, Cheongdam Branch, Seoul, Republic of Korea; 2 Korean Institute of Shelter Medicine, Seoul, Republic of Korea; 3 Department of Bio Life Science, Shingu College, Seongnam, Republic of Korea

**Keywords:** free-roaming cats, kitten proportion, longitudinal study, neutering rate, population dynamics, population structure, trap-neuter-return, urban ecology

## Abstract

**Background:**

Free-roaming cats (*Felis catus*) in urban environments are commonly managed through trap–neuter–return (TNR) programs, yet the long-term population effects of these measures remain inconsistently evaluated, particularly when considering both population size and structural dynamics.

**Objectives:**

This study aimed to assess long-term changes in free-roaming cat populations and population-structure patterns observed in the context of public TNR program in Seoul, Republic of Korea, using multiple demographic and structural indicators, specifically neutering rate, kitten proportion, and apparent retention.

**Methods:**

A longitudinal observational study was conducted across 17 fixed survey sites from 2017 to 2025. Repeated field surveys were performed biennially, with at least five visits per site. Population size, neutering rate, kitten proportion, and apparent retention were calculated via non-invasive photographic individual identification. Negative binomial and binomial mixed-effects models evaluated temporal trends and land-use differences. A logistic mixed effects model assessed apparent retention by baseline neutering status, and within-year encounter histories were used to estimate detection probability.

**Results:**

Overall population size and density showed a decreasing temporal pattern, while neutering rates generally increased and kitten proportions generally decreased. At the survey-year level, higher neutering rates coincided with lower kitten proportions. After adjustment for covariates and site-level clustering, cats neutered at baseline had significantly higher odds of apparent retention than non-neutered cats (OR = 3.06, 95% CI: 2.07–4.53). The pooled single-visit detection probability was 0.299, indicating that apparent retention remained subject to imperfect detection. In exploratory site-level comparisons, sites maintaining high neutering rates showed patterns consistent with lower kitten proportions and higher apparent retention, whereas sites with declining neutering rates showed patterns consistent with greater apparent turnover and higher kitten proportions.

**Conclusions:**

These findings describe population-structure patterns observed during implementation of Seoul's public TNR program, including reduced kitten counts and increased neutering probability over time. Given the observational nature of the study and the absence of untreated controls, these findings cannot demonstrate the causal effects of TNR. Nevertheless, structural indicators may complement simple procedure counts to identify sites requiring further intervention in urban cat management.

## 1 Introduction

Urban environments are increasingly affected by the presence of free-roaming cats (*Felis catus*), which are a source of persistent challenges such as noise, sanitation concerns, and community conflicts. Consequently, many countries have implemented policy interventions aimed at controlling free-roaming cat populations. Within this context, management strategies for free-roaming cats have shifted from reactive approaches focused on removal or complaint-driven actions toward structured population management based on neutering and long-term monitoring (1).

The Trap–Neuter–Return (TNR) approach has emerged as a central strategy in this transition. TNR involves the capture, surgical sterilization, and subsequent return of free-roaming cats to their original habitat, with the goal of suppressing reproduction and stabilizing population dynamics, ultimately reducing population size over time. However, recent studies have shown that the effectiveness of TNR is not determined solely by the number of surgeries performed, but is in fact strongly influenced by factors such as intervention intensity, spatial contiguity, and targeted implementation (1). High-intensity neutering applied across contiguous areas can result in measurable population declines, while targeted TNR in high-density or high-priority areas may contribute to improved management outcomes, such as reductions in shelter intake or kitten recruitment, when high local neutering coverage is maintained as an implementation requirement (2, 3). In high-conflict settings requiring immediate mitigation, however, TNR should not be regarded as a sufficient standalone response because its population-level effects occur gradually.

At the same time, TNR should be understood as one component of a broader population-management continuum rather than as a standalone intervention. Adoption provides the most direct reduction in population size for socialized or potentially socializable cats, whereas sterilization and return-based approaches may be appropriate for healthy free-roaming cats that are unlikely to benefit from long-term sheltering or adoption. Shelter-based return-to-field programs can reduce shelter intake pressure and avoid the euthanasia of otherwise healthy cats when sheltering does not provide a realistic pathway to adoption (4–7). In addition, low-cost or free sterilization and preventive veterinary services for owned or semi-owned cats may reduce future recruitment into free-roaming populations (8–12). Therefore, effective population management requires a combination of reproductive suppression, adoption where feasible, shelter-based decision-making, caregiver engagement, public education, and management of anthropogenic food resources. More broadly, because urban free-roaming cat populations are embedded in human-dominated resource systems, management outcomes may also be influenced by food-waste access, caregiver activity, abandonment, and other local socio-environmental factors (1).

One of the critical considerations in evaluating TNR effectiveness is that changes in population size alone are insufficient to fully explain population dynamics. Free-roaming cat populations are influenced by external factors such as immigration, abandonment, and compensatory reproduction driven by increased resource availability. Relying solely on population reduction as an indicator of success may therefore lead to misleading conclusions (1, 13). Recent studies have emphasized the need to incorporate multiple demographic indicators, including population abundance expressed as counts or density, neutering rate, kitten proportion, and observation-based persistence, to better understand population-level changes. However, outcomes of TNR remain context-dependent. Some studies have reported limited or inconsistent population-level effects, particularly when neutering intensity is insufficient, spatial coverage is incomplete, or immigration from surrounding areas continues (1, 13, 14). In contrast, more recent field programs have reported reductions in free-roaming cat populations, shelter intake, euthanasia, or complaints when sterilization was implemented intensively and sustained over time (2, 4–7, 15–17). These findings suggest that population-level outcomes depend strongly on local sterilization coverage, spatial targeting, intervention duration, and complementary management measures (1, 13–17). In addition, wildlife conservation perspectives have raised concerns regarding the ecological impacts of free-roaming cats, including predation on native species and broader ecosystem effects (18).

These contrasting findings highlight that TNR outcomes are context-dependent and influenced by implementation strategies, ecological conditions, and study design. Therefore, a comprehensive evaluation framework incorporating multiple demographic and structural indicators is essential for accurately assessing TNR effectiveness.

Among these indicators, the proportion of kittens reflects reproductive activity within the population, while the neutering rate indicates the degree of reproductive suppression. Furthermore, repeated identification of individuals across survey periods provides insight into population stability and the extent of external influx. Integrating these indicators is essential for understanding how TNR policies influence population structure and dynamics.

Various survey and analytical methods have been proposed to quantify free-roaming cat populations. Approaches such as line transect surveys and camera trapping, combined with statistical frameworks like spatially explicit capture–recapture, are commonly used to estimate population density while accounting for non-comprehensive detection (19–22). These methods are considered theoretically robust, as they facilitate correction for undetected individuals.

However, in high-density urban environments, such as Seoul, the application of these methods faces practical limitations. The effectiveness of camera trapping is constrained by privacy concerns and limited installation sites, while complex urban structures can reduce detection efficiency due to restricted visibility. Similarly, line transect surveys require consistent observation along predefined routes, which can be difficult to maintain in cities with irregular road networks and limited accessibility.

To address these constraints, this study employed a field-based survey approach designed to maximize the number of individuals observed within a defined area and time frame. By repeatedly surveying fixed locations, this approach enables the analysis of relative changes in population size and density over time, as well as population structure. Rather than estimating absolute population size, this study instead focuses on temporal trends and population dynamics derived from repeated observations.

Changes in free-roaming cat populations do not occur over short time scales, but are influenced by cumulative temporal and spatial factors. Longitudinal data based on repeated surveys of the same locations are therefore essential for evaluating the real-world effectiveness of TNR policies.

Seoul provides a valuable urban case for such analysis. Since 2008, the Seoul Metropolitan Government has implemented a publicly funded TNR program with increasing annual procedure numbers. According to official reports, the annual number of TNR procedures increased from approximately 5,800 in 2010 to over 10,000 after 2018, reaching approximately 14,000 by 2025, demonstrating the scale of municipal program input rather than, by itself, evidence of control efficacy or intensified population impact (23, 24). These city-wide procedure counts indicate the scale of program input, but they do not directly represent neutering coverage within specific territorial units. Indeed, increasing procedure numbers may also reflect persistent management demand due to remaining unneutered cats, ongoing reproduction, abandonment, immigration from surrounding areas, or incomplete spatial coverage. Because reproductive suppression depends not only on the number of procedures performed but also on the proportion of cats neutered within a defined local population, site-level indicators such as neutering rate, kitten proportion, and population turnover are required for program evaluation. Recent studies further emphasize that population-level outcomes depend on achieving substantial sterilization intensity within spatially defined target areas (2, 15–17). Whether intervention intensity at individual Seoul survey sites reached levels comparable to those reported in such programs could not be determined from the available city-wide procedure data. However, the present study was not designed to evaluate the effect of a high-intensity intervention within a specific target area. Rather, it aimed to characterize long-term population changes observed at fixed survey sites within Seoul under a city-wide, complaint-driven TNR system implemented over many years. Nevertheless, Seoul has conducted repeated monitoring of free-roaming cat populations, including population size, neutering status, and kitten occurrence, providing a rare long-term dataset at the metropolitan level.

In Korea, free-roaming cats are legally defined under the Animal Protection Act as animals that live independently in urban or residential areas and are excluded from mandatory rescue (25). Instead, local governments operate TNR-based management systems driven by citizen reports. This institutional framework prioritizes *in-situ* population management over removal. Furthermore, free-roaming cats in urban environments are generally known to maintain relatively limited home ranges, making repeated survey-based population analysis feasible (26, 27).

Although progress has been made, gaps in the literature persist. First, longitudinal studies repeatedly surveying the same urban areas under government-led TNR programs are limited. Second, prior studies focus on single indicators such as shelter intake or total population size, while integrated analyses incorporating population size, density, neutering rate, kitten proportion, and retention are scarce. Third, studies e valuating these factors within real-world urban policy contexts—particularly in large Asian cities—remain limited.

This study aimed to characterize long-term changes in free-roaming cat population structure observed across fixed survey sites in Seoul during implementation of a public TNR program. The analysis incorporated population abundance expressed as counts and density, neutering rate, kitten proportion, and apparent retention. By evaluating these indicators together, the study sought to provide an empirical framework for monitoring urban free-roaming cat populations and informing future management policies without attributing observed temporal changes to TNR alone.

## 2 Materials and methods

### 2.1 Study area, policy context, and study design

This study was conducted as a longitudinal observational evaluation of the population status and structural characteristics of free-roaming cats in Seoul, Republic of Korea. Seoul is a high-density metropolitan city with an area of approximately 605.6 km^2^, and a population exceeding 9.5 million. The city comprises heterogeneous land-use types, including residential, commercial, industrial, and green areas. This heterogeneity may influence the distribution, density, and movement patterns of free-roaming cats.

Since 2008, the Seoul Metropolitan Government has implemented a publicly funded TNR program, operating a complaint-driven TNR system across 25 administrative districts. Under this program, cats are captured following citizen reports, surgically sterilized, and returned to their original locations; ear-tipping is routinely performed to enable visual identification of neutered individuals, and vaccination against rabies has been included as part of the protocol since 2023. Caregivers or local residents may participate in trapping and release, and the program has been implemented as a one-stop process coordinated by local governments. This policy framework provides a basis for evaluating long-term changes in urban free-roaming cat populations through repeated observations. However, the present study was not intended to evaluate TNR in isolation. Seoul's free-roaming cat population dynamics may also have been influenced by broader urban management and human-associated factors, including shelter capacity, impoundment and shelter-intake practices, adoption systems, caregiver activity, intentional feeding, food-waste access and garbage management, abandonment, and local community responses to cats. Because standardized site-level and year-specific data on these factors were not available across all survey sites and years, they were treated as contextual factors and potential confounders but were not incorporated as covariates in the main longitudinal analyses.

The primary longitudinal surveys were performed biennially from 2017 to 2025 at 17 fixed survey sites. A preliminary exploratory survey was conducted in 2015 at nine sites to assess field feasibility and inform the subsequent sampling framework; however, these 2015 data were not included in the primary longitudinal analyses because the full 17-site design was implemented from 2017 onward. Each survey year was conducted between mid-September and October, the period when individuals born during the breeding season reach the juvenile stage, allowing simultaneous observation of both reproductive outcomes and the adult population.

### 2.2 Study sites and sampling framework

Study sites were selected based on land-use classifications defined under the National Land Planning and Utilization Act, and included four categories: residential, commercial, semi-industrial, and green areas (28). Site allocation was designed so as to broadly reflect the distribution of land-use types in Seoul, where residential areas account for the largest proportion, followed by green areas, with relatively smaller proportions of commercial and semi-industrial zones. Accordingly, more sites were assigned to residential areas (*n* = 11), while fewer sites were included for other land-use types (*n* = 2 each).

Site selection was informed by 2016 Seoul TNR implementation data. TNR density (cases per km^2^) was calculated at the administrative district level, and sites with densities approximating the citywide average (21.29 cases/km^2^) were preferentially selected to avoid overrepresentation of extreme conditions. This site-selection strategy was intended to improve comparability among survey sites, but it also narrowed the range of baseline intervention intensity available for analysis and may have attenuated the ability to detect dose–response relationships between TNR intensity and population outcomes.

Green areas were limited to urban parks and accessible green spaces, but excluded mountainous regions, to ensure comparability with other urban environments. Candidate sites within each land-use category were identified using map-based screening to match a standardized survey area (approximately 70,000–100,000 m^2^), corresponding to the area that could be systematically surveyed on foot within approximately 4 h. At two sites, the survey boundaries were modestly expanded from 2021 to include immediately adjacent areas in response to redevelopment; the monitoring locality, land-use classification, survey effort, and field procedures remained unchanged.

This approach was designed to capture structurally representative urban environments while ensuring the feasibility of repeated surveys and comparability of spatial coverage for longitudinal analysis, as opposed to a random sampling approach. A preliminary exploratory survey was conducted across nine sites in 2015 to assess field feasibility and inform the subsequent sampling framework. From 2017 onward, the survey design was restructured to include 17 fixed sites in order to better reflect land-use representation. Longitudinal analyses in this study were primarily based on data collected between 2017 and 2025, during which the study design remained consistent. The 2015 data were not included in the primary analytical dataset. Detailed site information is provided in Supplementary Table 1.

### 2.3 Field survey and individual identification

Field surveys were conducted at each site at least 5 times per survey year. Each survey lasted approximately 4 h and was carried out before sunset. Because surveys were conducted before sunset, the dataset primarily reflects cats visible during late daytime or crepuscular survey periods rather than the full 24-h activity cycle. This timing was kept consistent across survey years to improve comparability of repeated observations. Observers systematically traversed roads, alleys, and pedestrian pathways within each site to ensure comprehensive spatial coverage.

Survey routes and observation times were recorded using GPS-based tracking applications on mobile devices (e.g., Nike Run Club, Nike Inc Beaverton, OR, USA; Samsung Health, Samsung Electronics, Suwon-si, Gyeonggi-do, Republic of Korea) to maintain consistency in spatial coverage and survey effort across visits.

Observers with prior field survey experience were preferentially recruited to ensure data reliability. All observers underwent standardized training, including case-based exercises for individual identification. When identification was uncertain, decisions were made through joint review by experienced investigators, based on predefined identification criteria and contextual information (e.g., location, group association, and repeated observations), rather than the judgment of a single observer.

For each observed individual, the following variables were recorded: age class (adult or kitten), neutering status (based on ear-tipping), morphological characteristics (e.g., coat color, pattern, body shape, facial features, scars), and photographic records. Definitions and classification criteria are included in Table 1. Representative field recording sheets and photographic examples of individual identification are provided in the Supplementary material (Supplementary Figures 1, 2).

**Table 1:** Summary of variables recorded during field surveys and their definitions.

Variable	Definition
Survey round	Sequential visit number within a survey year (≥5 visits per site)
Age class	Adult or kitten (including juveniles), determined based on morphological characteristics
Neutering status	Determined by the presence of ear-tipping on the left ear
Morphological features	Coat color, pattern, tail shape, facial structure, scars, and other distinguishing characteristics
Photo records	Digital images of individuals with associated GPS coordinates for identification and verification

To support manual individual identification, a standardized recording chart (Supplementary Figure 1) developed by the research team was used, incorporating natural morphological features such as coat color, pattern distribution, eye color, tail morphology, facial structure, and other distinguishing characteristics. Individual identification was based on manual, non-invasive comparison of morphological characteristics and photographic records. This approach is relevant to feral cat monitoring, although previous work has shown that observer experience and image quality can influence individual identification from cat images (29). To improve consistency and minimize misidentification, decisions were made based on a combination of multiple features rather than a single characteristic. Ambiguous cases were resolved through consensus review among trained observers and experienced investigators.

To quantify and account for imperfect detection during this observational process, within-year binary encounter histories were constructed from repeated survey visits (at least five visits per year) for each individually identifiable cat. Because individuals with all-zero encounter histories could not appear in the dataset, the single-visit detection probability (*p*) was estimated using a likelihood conditional on at least one detection. For standardized comparison, the cumulative probability of at least one detection over five visits was calculated as 1–(1–p)^∧^5. Site-year estimates were treated as supplementary because several cells had small sample sizes.

For longitudinal re-identification, ear-tip status was not used as the primary matching criterion. Individuals were first matched using morphology-based features, including coat color, pattern distribution, facial markings, tail morphology, body shape, scars, and other distinctive characteristics. Ear-tip status was recorded as an indicator of neutering status, but it was not considered sufficient on its own to confirm individual identity. This was particularly important because some individuals that were not ear-tipped in an earlier survey were re-identified as ear-tipped in a later survey after undergoing TNR between survey periods. Individuals with insufficiently distinctive morphology, such as uniformly colored cats without reliable additional features, were excluded from apparent retention analyses or treated as uncertain cases. Due to uncertainty arising from factors such as rapid movement, distant observation, or uniform coat color, individual identification was not always certain. To account for this, both minimum and maximum counts were estimated. The minimum count assumed that uncertain individuals were previously observed individuals (i.e., duplicates), whereas the maximum count treated them as new individuals. The mean of these two values was defined as the mean count and used for subsequent analyses. The use of minimum and maximum counts was intended to provide a conservative estimation range that accounts for identification uncertainty in observational studies. Although this identification approach was developed specifically for this study, it was designed to ensure consistency and reproducibility through standardized criteria and observer training.

### 2.4 Population metrics and longitudinal analysis

To evaluate population structure and dynamics, the following indicators were calculated: population abundance, expressed as counts and area-standardized density, neutering rate, kitten proportion, and individual persistence, expressed as apparent retention (i.e., the proportion of individuals observed in an earlier survey period that were re-identified in the same survey site during the subsequent survey period, reflecting observation-based persistence within the study site).

Kittens were distinguished from adults based on morphological criteria, with juveniles (approximately 5–6 months old) being included in the kitten category (30). The kitten proportion was calculated as the number of kittens divided by the mean population count.

Neutering status was determined based on ear-tipping, and the neutering rate was calculated as the proportion of neutered individuals relative to the mean population count.

For both the descriptive status-specific comparisons and the mixed-effects analysis, neutering status was assigned according to the earlier survey of each interval (baseline status). Cats that were not ear-tipped at baseline but were re-identified as ear-tipped at the subsequent survey were retained in the baseline non-neutered category. Their later ear-tip status was recorded to document that TNR had occurred during the interval but was not used to reclassify baseline neutering status.

Apparent retention was defined as the proportion of individually identifiable cats observed at an earlier survey that were re-identified at the same survey site during the subsequent survey. This observation-based metric does not distinguish continued presence from temporary absence, emigration, mortality, or failure to detect a cat that remained present. Let N(t−1) and N(t) denote the numbers of individually identifiable cats included in the re-identification dataset at the earlier and subsequent surveys, respectively, and let D denote the number observed at both surveys. The following indices were calculated:


$$\begin{array}{l} \text{Apparent retention rate }\left(\text{\%}\right)=\left[\text{D}/\text{N}\left(\text{t}-1\right)\right]\;\times 100\\ \text{Apparent new observation proportion }\left(\text{\%}\right)=\\ \left[\left(\text{N}\left(\text{t}\right)\;-\text{ D}\right)/\text{N}\left(\text{t}\right)\right]\;\times 100\\ \text{Apparent non}-\text{retention proportion }\left(\text{\%}\right)\;=\\ \left[\left(\text{N}\left(\text{t}-1\right)\;-\text{ D}\right)/\text{N}\left(\text{t}-1\right)\right]\;\times 100 \end{array}$$

These indices describe changes in the observed composition of the population and should not be interpreted as direct measures of survival, mortality, immigration, emigration, or true site fidelity.

### 2.5 Statistical analysis

Statistical analyses were performed using R (version 4.3.2; R Foundation for Statistical Computing, Vienna, Austria) to evaluate temporal trends, structural shifts, and habitat-specific differences while accounting for repeated observations across fixed survey sites.

For count outcomes, including adult free-roaming cat counts (rounded mean counts) and total kitten counts per site, generalized linear mixed models (GLMMs) were applied. Although a Poisson distribution (log link) was initially considered, negative binomial models were ultimately used for final inference because they demonstrated superior model fit when evaluated using information criteria and simulation-based residual diagnostics. To evaluate overall temporal trends, survey year was treated as a continuous variable, and land-use type was included as a fixed effect. Survey site was specified as a random effect in all models to control for large between-site variations and potential sampling noise. Effects for count data are reported as incidence rate ratios (IRRs) with 95% confidence intervals (Cis). To evaluate whether the rate of population change differed by habitat type, an interaction term between the continuous year variable and land-use type was incorporated into the population model.

For the analysis of annual proportions, specifically neutering status, a binomial GLMM (logit link) was fitted to model successes vs. failures. In this model, survey year was treated as a categorical factor to estimate year-specific parameters, with land-use type included as a fixed effect and survey site as a random effect.

Apparent retention at the subsequent survey was additionally analyzed as a binary outcome using a binomial logistic mixed-effects model. Baseline neutering status was the primary explanatory variable. The model adjusted for baseline age class (adult or kitten), land-use category (residential or non-residential), and survey interval, and included a site-specific random intercept to account for clustering of individuals within survey sites. Of the 865 interval-level observations, 851 with known baseline neutering status were included in the adjusted analysis. The 14 observations with unknown baseline neutering status were excluded because all were classified as not retained, resulting in complete separation when unknown status was modeled as a separate category. The three observations with unknown baseline age class were a subset of these excluded observations. Sex and baseline health status were not available in the analytic dataset and therefore could not be included as covariates. Associations are reported as odds ratios (Ors) with 95% confidence intervals and should not be interpreted as causal effects.

*Post-hoc* comparisons and estimated marginal means were calculated and back-transformed from the logit or log scales to the probability or count scales using the emmeans package. Results are reported as model-predicted probabilities or IRRs with corresponding 95% Cis. Statistical significance was defined as *p* < 0.05. The 2017–2025 dataset was used as the primary standardized analysis because the survey framework was consistent across these years, whereas the 2015 exploratory survey data were evaluated separately as a sensitivity analysis.

### 2.6 Additional analyses and ethical considerations

Additional analyses were conducted by comparing data collected in 2023 and 2025 to identify sites exhibiting decreased neutering rates or increased kitten proportions, as well as sites that maintained stable population structures. For these sites, contextual factors such as local TNR implementation intensity and the distribution of feeding stations were further examined.

This study involved only non-invasive observation and photography conducted in publicly accessible areas. No animals were captured or handled during the surveys. This study did not involve experimental manipulation or direct interaction with animals and, therefore, did not require ethical approval under applicable regulations. Throughout the study, efforts were taken to minimize disturbance to both animals and local residents.

## 3 Results

### 3.1 Overall temporal trends in free-roaming cat population

The combined survey area was approximately 1.49 km^2^ in 2017 and 2019 and 1.53 km^2^ from 2021 to 2025, reflecting minor boundary adjustments at two sites. Across the 17 fixed survey sites, the descriptive total mean count of free-roaming cats declined from 334.0 in 2017 to 261.0 in 2025, corresponding to an overall observed decrease of 21.9%. When expressed as a descriptive annualized metric over the 8-year interval, this represented an approximate decline of 3.0% per year. The corresponding area-standardized density declined from 223.5 to 170.9 cats/km^2^ (Supplementary Table 2).

To formally evaluate these temporal changes while accounting for repeated observations across sites, GLMMs were fitted for the primary standardized dataset from 2017 to 2025 (Table 2). For adult free-roaming cat counts, the negative binomial GLMM provided a substantially better fit than the Poisson model based on information criteria. The model estimated a modest downward annual association in adult cat counts, corresponding to an incidence rate ratio (IRR) of 0.9676 per year (95% CI: 0.9343–1.0021; *p* = 0.0656). This estimate indicated a downward direction but did not reach statistical significance, and the confidence interval included no change.

**Table 2:** Model-based analyses of temporal trends and land-use differences in free-roaming cat population indicators from 2017 to 2025.

Outcome	Model	Predictor	Estimate	SE	Test statistic	*p*-value	Effect size	95% CI
Adult cat count	Negative binomial GLMM	Year	−0.0329	0.0179	*z* = −1.841	0.0656	IRR = 0.9676	0.9343–1.0021
Adult cat count	Negative binomial GLMM	Residential land use	0.4072	0.2539	*z* = 1.604	0.1088	IRR = 1.5026	0.9135–2.4715
Kitten count	Negative binomial GLMM	Year	−0.2599	0.0478	*z* = −5.439	< 0.001	IRR = 0.7711	0.7022–0.8468
Kitten count	Negative binomial GLMM	Residential land use	0.0168	0.2817	*z* = 0.059	0.9526	IRR = 1.0169	0.5854–1.7664
Neutering status	Binomial GLMM	2019 vs. 2017	0.3640	0.2123	*z* = 1.715	0.0864	OR = 1.4391	0.9493–2.1817
Neutering status	Binomial GLMM	2021 vs. 2017	1.5232	0.2044	*z* = 7.452	< 0.001	OR = 4.5871	3.0729–6.8474
Neutering status	Binomial GLMM	2023 vs. 2017	2.5060	0.2120	*z* = 11.821	< 0.001	OR = 12.2555	8.0886–18.5688
Neutering status	Binomial GLMM	2025 vs. 2017	2.2372	0.2115	*z* = 10.578	< 0.001	OR = 9.3670	6.1883–14.1784
Neutering status	Binomial GLMM	Residential land use	0.1955	0.3885	*z* = 0.503	0.6148	OR = 1.2159	0.5678–2.6039

GLMM, generalized linear mixed model; IRR, incidence rate ratio; OR, odds ratio; CI, confidence interval. Survey site was included as a random effect. For count outcomes, negative binomial GLMMs were used after model comparison with Poisson models. For neutering status, a binomial GLMM was used, with 2017 as the reference year. Residential land use was compared with non-residential land use as the reference category.

For kitten counts, the negative binomial GLMM showed a significant decline over time. The estimated annual IRR was 0.7711 (95% CI: 0.7022–0.8468; *p* < 0.001), corresponding to an estimated annual decrease of approximately 22.9% in observed kitten counts.

The binomial GLMM for neutering status showed a substantial increase in model-predicted neutering probability over the study period. Compared with the 2017 baseline, the odds of being neutered were not significantly different in 2019, but were significantly higher from 2021 onward. The model-predicted neutering probability increased from 14.0% in 2017 to 42.7% in 2021, peaked at 66.5% in 2023, and remained elevated at 60.3% in 2025; year-specific predicted probabilities and 95% confidence intervals are provided in Supplementary Table 6.

Reflecting these modeled trends, the observed neutering rate also descriptively increased over time, reaching its highest level in 2023, followed by a slight decrease in 2025. In contrast, kitten proportion showed an overall decreasing trend, reaching its lowest level in 2023, followed by a modest increase in 2025. Across the five aggregated survey years, neutering rate and kitten proportion showed an inverse descriptive pattern (Figure 1; Supplementary Table 2). Because both measures were calculated as proportions of the mean count, this relationship may partly reflect compositional constraints and was therefore not interpreted as independent evidence of a causal effect.

**Figure 1:**
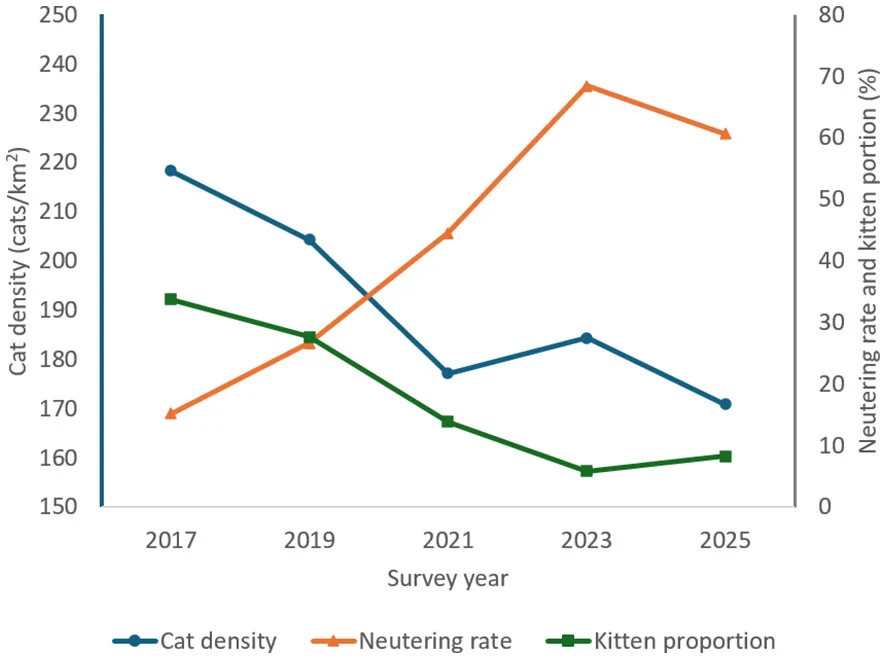
Temporal changes in population indicators of free-roaming cats from 2017 to 2025. Cat density is shown on the left y-axis, whereas mean site-level neutering rate and kitten proportion across the 17 fixed survey sites are shown on the right y-axis. Values are descriptive survey-year summaries; site-level temporal changes were evaluated using mixed-effects models, and the inverse pattern between neutering rate and kitten proportion should not be interpreted causally because the two measures share the same denominator.

### 3.2 Comparison between residential and non-residential areas

Differences in population abundance and structure between residential and non-residential areas were evaluated over the study period (Figure 2; Supplementary Table 3). Descriptively, cat density was generally higher in residential areas than in non-residential areas across most survey years, although the magnitude of this difference varied over time. Both area types showed declining density trends, increasing neutering rates, and decreasing kitten proportions during the study period.

**Figure 2:**
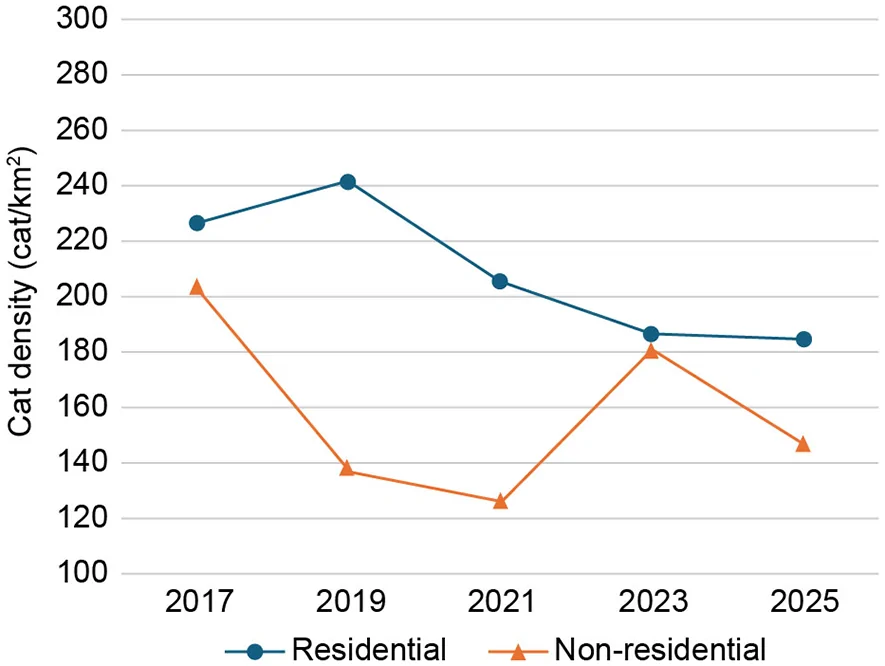
Temporal changes in cat density (cats/km^2^) in residential and non-residential areas from 2017 to 2025. Cat density was descriptively higher in residential areas in most survey years; however, mixed-effects analyses did not support a statistically significant land-use effect or a difference in temporal slopes between land-use categories.

However, the GLMMs indicated that land-use type was not significantly associated with any of the primary outcomes (Table 2). Specifically, there were no significant differences between residential and non-residential sites for adult cat counts (IRR = 1.5026, 95% CI: 0.9135–2.4715; *p* = 0.1088), kitten counts (IRR = 1.0169, 95% CI: 0.5854–1.7664; *p* = 0.9526), or neutering probability.

Furthermore, an interaction model showed no evidence that the temporal trend differed by land-use type (Supplementary Table 7). The interaction between survey year and residential land use was not statistically significant (IRR = 0.9994, 95% CI: 0.9280–1.0764; *p* = 0.9883). *Post-hoc* slope estimates were similar for non-residential sites (IRR = 0.9680, 95% CI: 0.9112–1.0283) and residential sites (IRR = 0.9674, 95% CI: 0.9267–1.0099), indicating that the observed rate of change did not differ meaningfully between land-use categories. Descriptively, cat density was higher in residential areas than in non-residential areas in most survey years; however, the model-based analysis did not support a statistically significant land-use effect. Therefore, these apparent residential–non-residential differences should be interpreted cautiously and may reflect sampling variation among sites rather than a definitive land-use effect.

### 3.3 Detection probability and apparent retention across survey intervals

A total of 1,478 individual within-year encounter histories were included in the detection analysis. This number represents all unique individuals observed at least once across visits, which differs slightly from the sum of the estimated mean counts (*n* = 1,460) due to the inclusion of transient observations and the mathematical derivation of the mean abundance metric. The pooled conditional single-visit detection probability was 0.299 (95% CI: 0.287–0.312). Year-specific estimates ranged from 0.262 (95% CI: 0.235–0.288) in 2017 to 0.344 (95% CI: 0.314–0.374) in 2025. Under a standardized five-visit calculation, the cumulative probability of detecting a present individual at least once ranged from approximately 78.1% to 87.8%, corresponding to a probability of remaining undetected throughout all five visits of approximately 12.1% to 22.0% (Supplementary Table 4). Thus, repeated visits increased cumulative detection, but did not eliminate the possibility that a cat remaining present was not observed.

Apparent retention was evaluated based on the re-identification of individuals between consecutive survey periods across the 17 fixed survey sites. Based on individually identifiable cats included in the interval-level re-identification dataset, both the number and proportion of cats re-identified at the same survey site increased across the three survey intervals, indicating an overall increase in repeated observations over time. Descriptively, cats classified as neutered at baseline showed higher apparent retention than those classified as non-neutered at baseline (Figure 3; Supplementary Table 5A). Because a failure to re-identify an individual could reflect departure from the site, mortality, or imperfect detection of a cat that remained present, these values are reported as apparent retention rather than true retention or site fidelity.

**Figure 3:**
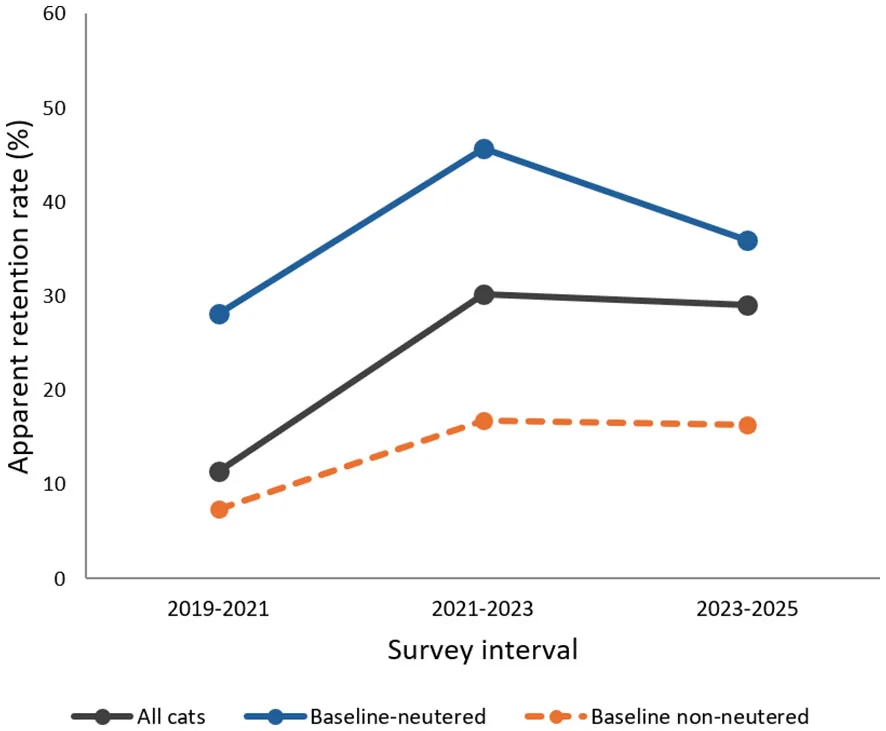
Apparent retention of individually identifiable free-roaming cats across consecutive survey intervals. Neutering status was assigned at the earlier survey of each interval. Cats that were non-neutered at baseline but underwent TNR before the subsequent survey remained classified in the baseline non-neutered group for this comparison. Values shown are descriptive; adjusted associations are presented in Supplementary Table 5B.

Among 851 interval-level observations with known baseline neutering status, cats that were neutered at baseline had significantly higher odds of apparent retention at the subsequent survey than cats that were non-neutered at baseline, after adjustment for baseline age class, land-use category, and survey interval, with survey site included as a random intercept (OR = 3.06, 95% CI: 2.07–4.53; *p* < 0.001). Kittens had lower odds of apparent retention than adults (OR = 0.34, 95% CI: 0.16–0.74; *p* = 0.006), whereas land-use category was not significantly associated with apparent retention (OR = 0.94, 95% CI: 0.58–1.52; *p* = 0.795). Compared with the 2019–2021 interval, the odds of apparent retention were significantly higher in 2021–2023 (OR = 2.20, 95% CI: 1.39–3.49; *p* < 0.001). The corresponding difference for 2023–2025 did not reach conventional statistical significance (OR = 1.61, 95% CI: 0.998–2.60; *p* = 0.051; Supplementary Table 5B).

### 3.4 Exploratory comparison of selected sites showing stable or vulnerable population structures (2023–2025)

Among the 17 survey sites, seven sites showing notable descriptive changes in population structure between 2023 and 2025 were selected for exploratory comparison. These sites were classified *post hoc* as sites with relatively stable population structures (S01, S04, S05, S11), where high neutering rates and low kitten proportions were maintained, and sites with vulnerable population structures (S02, S07, S12), where neutering rates decreased or kitten proportions increased (Table 3).

**Table 3:** Exploratory comparison of population structure and apparent retention in selected survey sites (2023–2025).

Site category	Site	Cats, 2023	Neutering, 2023 (%)	Kittens, 2023 (%)	Cats, 2025	Neutering, 2025 (%)	Kittens, 2025 (%)	Re-identified (D)	Retention (%)	Neutered among re-identified (%)	New observations (%)
Stable	S01	24	91.7	0.0	23	95.7	0.0	13	54.2	95.0	43.5
	S04	13	76.9	0.0	14	85.7	0.0	7	53.9	100.0	50.0
	S05	23	91.3	0.0	17	82.4	0.0	7	30.4	100.0	58.8
	S11	10	50	20.0	6	100.0	0.0	5	50.0	100.0	16.7
	Mean		77.5	5.0		90.9	0.0		47.1	98.8	42.2
Vulnerable	S02	14	71.4	7.1	15	33.3	13.3	3	21.4	33.3	80.0
	S07	11	63.6	0.0	25	40.0	20.0	5	45.5	100.0	80.0
	S12	6	66.7	0.0	10	20.0	50.0	0	0.0	–	100.0
	Mean		67.2	2.4		31.1	27.8		22.3	66.7	86.7

Retention rate (%) was calculated as (D/N_(2023)_) × 100, where D is the number of individuals re-identified in 2025 and N_(2023)_ is the number observed in 2023. New observation proportion (%) was calculated as [(N_(2025)_ – D)/N_(2025)_] × 100. Site categories were assigned *post hoc* for exploratory illustration, and mean values are presented for each category. A dash (–) indicates that the value could not be calculated.

Available contextual information was reviewed for the selected sites, including changes in feeding-site number and use, surrounding land-use characteristics, nearby TNR implementation, timing of neutering relative to the breeding season, and potential movement between adjacent areas. In some sites, feeding sites continued to be used by cats, including kittens, suggesting that these locations may function as focal activity areas for reproductively active cats. In addition, TNR conducted in nearby but spatially connected areas may have influenced site-level estimates of neutering rate. Because these factors were not collected systematically across all survey sites and years, they were used only to contextualize site-level differences and were not included as covariates in the exploratory comparison.

In stable management sites, the mean neutering rate increased from 77.5% in 2023 to 90.9% in 2025, while the kitten proportion decreased from 5.0 to 0.0%. The apparent retention rate was 47.1%, and 98.8% of re-identified individuals were neutered. These values describe the observed composition of the selected sites but remain subject to imperfect and potentially differential re-identification.

In contrast, vulnerable management sites showed a decrease in neutering rate from 67.2% in 2023 to 31.1% in 2025, accompanied by an increase in kitten proportion from 2.4 to 27.8%. The apparent retention rate was lower (22.3%), and the proportion of neutered individuals among re-identified cats was also lower (66.7%).

The proportion of newly observed individuals was markedly higher in vulnerable sites (86.7%) than in stable sites (42.2%). This pattern was consistent with greater apparent turnover in the selected vulnerable sites but does not distinguish immigration from mortality, emigration, imperfect detection, or other processes.

## 4 Discussion

This study evaluated long-term changes in free-roaming cat populations and their structural characteristics through repeated surveys conducted at 17 sites in Seoul from 2017 to 2025. The generalized linear mixed model (GLMM) analyses support a nuanced interpretation of these long-term monitoring results. Although the descriptive total mean number of cats and population density showed a decreasing pattern over time, the formal model for adult cat counts indicated only a downward estimate that did not reach statistical significance. In contrast, the most robust population-structure changes observed during the study period were a significant decline in kitten counts and a substantial increase in model-predicted neutering probability after 2021. Until 2023, the observed neutering rate increased while the kitten proportion declined. However, this inverse year-level pattern was based on only five aggregated survey-year observations and was treated as descriptive. The site-level mixed-effects analyses showed a significant temporal decline in kitten counts and a substantial increase in neutering probability after 2021. These concurrent trends were consistent with changes in population structure but should not be interpreted as evidence that TNR alone caused the observed changes.

These temporal patterns should be interpreted within the broader human and resource context of Seoul, rather than as the isolated effect of TNR alone. Factors such as intentional feeding, food-waste access, shelter availability, abandonment, caregiver activity, shelter intake and adoption practices, and community responses may also have influenced local population dynamics. Because standardized site-level data on these factors were not available across all survey sites and years, the observed associations between neutering coverage and population-structure indicators should be interpreted as patterns observed under Seoul's real-world urban management context, not as evidence that TNR alone caused all observed population changes.

A key contribution of this study is that it evaluated population management outcomes using multiple indicators rather than relying solely on changes in population size. Although the overall population size declined over time, the slight decrease in neutering rate and increase in kitten proportion observed in 2025 relative to 2023 indicate that population size alone is insufficient to characterize management stability. Internal population structure and turnover dynamics must also be considered. Even with similar population sizes, some selected sites showed high neutering rates, low kitten proportions, and relatively high apparent retention, whereas others showed declining neutering rates, increased kitten proportions, and higher proportions of newly observed individuals. Available contextual information suggested that the timing and spatial distribution of TNR may have differed among these sites; however, these factors were not collected systematically and cannot be identified as causes of the observed patterns. Given that TNR implementation in Seoul is largely complaint-driven, the findings support continued monitoring and coordinated follow-up across adjacent areas rather than demonstrating that any particular intervention schedule produced the observed site-level differences.

This study also demonstrates that repeated surveys within a limited spatial extent can improve the reliability and interpretability of population monitoring. Similar longitudinal monitoring principles have been applied to free-roaming animal management elsewhere. Repeated standardized route counts have been used to track street-dog population changes in Jaipur, India (31), while recent Australian cat management research similarly incorporated repeated standardized street counts to evaluate changes in free-roaming cat density (17). This type of repeated standardized monitoring is particularly useful in urban settings, where free-roaming cat populations are difficult to comprehensively census because individuals may be hidden or temporarily unobserved. In this study, each site was surveyed at least five times, and individual cats were identified using photographic records and morphological characteristics. In addition, minimum, maximum, and mean counts were estimated to account for uncertainty in individual identification. As demonstrated by the detection analysis, repeated within-year surveys reduced, but did not eliminate, uncertainty arising from imperfect detection. The pooled conditional single-visit detection probability was 0.299, corresponding to a standardized five-visit cumulative detection probability of approximately 83.1%. Year-specific cumulative detection probabilities ranged from approximately 78.1%−87.8%, indicating that approximately 12.2% to 21.9% of cats present under the model assumptions could remain undetected across five visits.

These detection estimates should nevertheless be interpreted cautiously. The analysis assumed constant detection probability within each pooled or year-specific model; visit-specific differences in survey effort could not be modeled explicitly, and demographic closure during each within-year survey period could not be independently verified. Survey timing may also have influenced detection, because surveys were conducted before sunset and may therefore have underdetected individuals with predominantly nocturnal or wide-ranging activity patterns. Together, these findings support use of the term apparent retention: some cats classified as not retained may have remained within the survey site but were not observed. Accordingly, apparent retention should be interpreted as observation-based persistence under standardized survey conditions, rather than as direct evidence of true site fidelity, survival, or movement behavior. These estimates quantify detection uncertainty but do not constitute formal capture–recapture estimates of abundance, and the overall results should be interpreted as reflecting relative changes and trends in population dynamics rather than absolute population estimates.

Although the inverse year-level pattern between neutering rate and kitten proportion is biologically consistent with reproductive suppression following TNR, it should be interpreted cautiously. This relationship was based on only five aggregated survey-year observations, and both variables were calculated using the mean count as the denominator. Because these indicators are compositional proportions within the same observed population, part of the inverse relationship may reflect the mathematical structure of the data rather than an entirely independent demographic process. Therefore, this year-level pattern was treated as descriptive and was not interpreted as causal evidence by itself. Instead, it should be considered alongside other structural indicators, including changes in population density, apparent retention, and the proportion of newly observed individuals. The apparent retention rate, derived from repeated identification of individuals across survey periods, represents an observation-based indicator of population persistence. Neutered cats were more frequently re-identified than non-neutered cats across all intervals. One possible biological explanation for this pattern is that neutering may reduce reproductive and agonistic behaviors in free-roaming cats, which could contribute to repeated observation within the same area (32).

At the same time, baseline neutering status may also reflect pre-existing differences among cats. In a complaint-driven TNR system, established or caregiver-associated cats may be more likely to have been neutered before the baseline survey and may also be more likely to be re-observed at the same site. Therefore, the association between baseline neutering status and apparent retention may partly reflect such selection and should not be interpreted as a causal effect of neutering.

This interpretation should be made cautiously because apparent retention is not a direct measure of survival, movement, or site fidelity. A potential residual limitation is that re-identification probability may still have differed according to neutering status because ear-tipped cats carry a permanent visual mark. However, ear-tip status was not used as the primary criterion for longitudinal re-identification in this study. Individuals were matched primarily using morphology-based features, including coat color, pattern distribution, facial markings, tail morphology, body shape, scars, and other distinctive characteristics. Individuals with insufficiently distinctive morphology were excluded from apparent retention analyses or treated as uncertain cases. Therefore, although differential re-identification probability cannot be completely ruled out, the observed difference in apparent retention is unlikely to be explained solely by the presence of ear-tipping. Comparisons of apparent retention by neutering status should therefore be interpreted as observation-based persistence patterns rather than as direct evidence of behavioral differences or true site fidelity.

The numerically higher densities observed in residential areas may be consistent with greater availability of anthropogenic resources, including intentional feeding, food waste, and shelter provided by urban structures. Previous studies have reported that urban free-roaming cats may occupy relatively small home ranges and aggregate in resource-rich environments, supporting this interpretation as plausible ecological context (27, 33, 34). However, although residential sites showed higher descriptive densities in several survey years, land-use type was not statistically significant in the model-based analysis. Therefore, the apparent residential–non-residential difference should not be interpreted as a definitive land-use effect. While anthropogenic food resources and shelter availability may provide ecological context for urban cat distribution, the present data do not demonstrate that these factors explain the observed density patterns. Additionally feeding-station data were available only for the 2023–2025 contextual sub-analysis of selected sites and were not available as standardized covariates across all survey sites and years. Therefore, feeding stations and other food-resource variables could not be formally evaluated as independent predictors in the main land-use analysis. The comparison between stable and vulnerable management sites was retained as an exploratory, descriptive sub-analysis to illustrate site-level heterogeneity in population-structure indicators. Stable sites showed increased neutering rates (from 77.5 to 90.9%), reduced kitten proportions (from 5.0 to 0.0%), and relatively high retention rates (47.1%). In contrast, vulnerable sites exhibited decreased neutering rates (from 67.2 to 31.1%), increased kitten proportions (from 2.4 to 27.8%), and lower retention rates (22.3%), along with substantially higher proportions of newly observed individuals. These patterns were consistent with an association between high neutering coverage, suppressed reproduction, and more stable observed population structures, whereas areas with reduced neutering coverage showed patterns consistent with increased turnover and population instability (35). However, these relationships should not be interpreted as causal effects of TNR alone, because site-level differences may also have been influenced by feeding-site use, caregiver activity, food-waste access, abandonment, movement between adjacent areas, shelter intake and adoption practices, and local community responses. Therefore, these findings support the use of structural indicators to identify sites requiring follow-up management, rather than demonstrating that spatially targeted TNR alone produces stable population outcomes. In addition, recent meta-research has suggested that the TNR literature may be influenced by citation networks, in which studies preferentially cite findings that support similar conclusions (36). This may contribute to inconsistencies in reported outcomes and highlights the importance of evaluating TNR effectiveness using diverse methodological approaches and multiple population-level indicators, as applied in the present study.

The temporal patterns observed in this study are consistent with an association between sustained, high-coverage neutering and more stable population structure. The concurrent non-significant downward estimate in adult counts, substantial increase in neutering rate, significant reduction in kitten counts, and higher apparent retention among neutered cats suggest that long-term TNR implementation may be linked to changes in urban free-roaming cat population structure. However, because this study did not include untreated control areas or a counterfactual scenario, these patterns should not be interpreted as definitive evidence of program causation.

The 2019–2021 interval coincided with the COVID-19 pandemic. However, Seoul's municipal TNR program was not substantially interrupted during this period. Annual TNR procedures increased from 11,775 in 2019 to 12,047 in 2020, 13,131 in 2021, and 14,179 in 2022. Thus, a pandemic-related reduction in TNR activity is unlikely to explain the 2021 findings. Nevertheless, other pandemic-related changes in human activity or cat population dynamics cannot be excluded.

The land-use analysis should be interpreted cautiously. Commercial, semi-industrial, and green areas were represented by only two sites each and were therefore combined into a single non-residential category. This limited statistical power and may also have obscured differences among these heterogeneous land-use types. Accordingly, the absence of a significant residential–non-residential effect should not be interpreted as evidence that land use has no influence on free-roaming cat population structure.

Furthermore, local governments in Korea, including Seoul, maintain systematic records of TNR activities, including capture locations, timing, and individual information such as photographs (24). These data provide a valuable foundation for identifying areas with high complaint rates or increased shelter intake, particularly for kittens. Such information can support a shift from quantity-based operations to data-driven, spatially strategic management, including targeted TNR and complementary interventions in high-priority areas. Successful examples from other contexts further illustrate the potential value of integrated, non-lethal management approaches. For instance, a socio-ecological strategy implemented on a protected island with human residents demonstrated that non-lethal management of free-roaming cats can contribute to biodiversity conservation when combined with sustained intervention and stakeholder engagement (37). However, this example has limited transferability to Seoul because a small protected island differs substantially from a large open metropolis of approximately 9.5 million residents in spatial scale, population openness, governance structure, and patterns of cat movement and abandonment. Therefore, it is cited only as a contextual example supporting sustained, coordinated, and socially supported management, rather than as direct evidence for comparable outcomes in Seoul. Consistent with previous reviews, sterilization should be considered one component of integrated cat population management and may need to be combined with complementary measures such as adoption, shelter-based interventions, caregiver engagement, kitten management, and management of anthropogenic food resources (38).

Despite these observed patterns, several limitations of the current TNR policy context were also identified. The slight decrease in neutering rate and increase in kitten proportion observed in 2025 suggest that maintaining high neutering coverage over time is challenging. In the exploratory vulnerable-site comparison, lower apparent retention and higher proportions of newly observed individuals were consistent with renewed population instability, but these patterns cannot be attributed to insufficient or inconsistent TNR alone. Urban free-roaming cat populations are open systems, and immigration from surrounding areas, abandonment, resource availability, and imperfect detection may offset or obscure local sterilization efforts.

Operational constraints may also slow reproductive suppression. As neutering coverage increases, the remaining unneutered individuals may become more difficult to capture, reducing trapping efficiency. Under Korean TNR guidelines, pregnant or lactating females and cats below specified body weight thresholds (e.g., < 2 kg) are subject to eligibility restrictions (39), which may allow some reproductively active individuals to remain temporarily in the population. The present study does not advocate indiscriminate surgery or the lowering of veterinary safety standards for cats that are not appropriate surgical candidates. Rather, it highlights the need for individualized veterinary risk–benefit assessment within community cat TNR programs. Clinical management texts and veterinary spay-neuter guidelines emphasize that future opportunities for care may be limited once a free-roaming cat is released, making the risk–benefit balance of neutering different from that of owned cats (40, 41). Published veterinary guidelines indicate that cats undergoing elective surgery should be in reasonably good health and body condition, but that conditions such as pregnancy, estrus, thin body condition, or mild upper respiratory disease do not necessarily preclude safe neutering when appropriate anesthetic, surgical, and perioperative care are provided (41). Early-age neutering before sexual maturity is also strongly recommended and can be safely performed from an early age under appropriate protocols (36, 37). Therefore, reproductive suppression should be pursued not by weakening safety standards, but by improving the timing, spatial coverage, and veterinary decision-making of TNR, including targeted implementation before peak breeding periods, repeated follow-up in vulnerable areas, and early identification and foster-based management of kittens.

Taken together, these findings describe a gradual population decline and changes in population structure observed during implementation of Seoul's TNR program. The modest magnitude of decline and the absence of untreated controls indicate that TNR should not be characterized as a sufficient or efficient standalone approach. Within integrated management, spatially focused and proactively timed neutering may remain an operational component, alongside adoption, shelter-based interventions, caregiver engagement, kitten management, and management of anthropogenic food resources (15).

In conclusion, this study describes long-term changes in free-roaming cat population size and population-structure indicators across fixed survey sites in Seoul in the context of a public TNR program. During the study period, the observed indicators, including total cat counts, neutering rate, kitten proportion, and apparent retention, showed temporal patterns consistent with changes in population structure. However, because this was an observational study without untreated control sites or counterfactual comparisons, these findings should not be interpreted as direct causal evidence of TNR policy effects. Instead, they provide an observation-based framework for understanding how multiple population indicators can be monitored together over time.

Therefore, future management strategies should move beyond simple counts of surgical procedures and incorporate structural indicators such as neutering coverage, reproductive output, and population turnover, together with complementary measures such as adoption, shelter-based interventions, caregiver engagement, and management of anthropogenic food resources. From a policy perspective, these findings support a reasoned shift away from evaluating TNR programs solely by the number of procedures performed. Although causal effects cannot be established from the present observational design, program evaluation may be strengthened by incorporating structural indicators such as neutering coverage within defined areas, kitten proportion, and population turnover. Linking funding decisions or program evaluation to such indicators should therefore be considered as a policy approach, rather than as a direct conclusion demonstrated by this study.

## Data Availability

The anonymized site-level dataset supporting the findings of this study is provided in Supplementary Table 1. The dataset includes annual site-level summary counts and derived indicators used in the analyses, including survey year, anonymized site ID, land-use category, survey area, mean cat count, cat density, neutered cat count, and kitten count. To reduce potential risks to free-roaming cats and caretakers, raw photographs, individual-level photographic matching records, precise GPS coordinates, colony locations, and feeding-station locations are not publicly released. Survey sites are identified only by anonymized site codes and broad land-use categories.
