# Supplementary material for: Beyond population size: long-term changes in free-roaming cat population structure during TNR implementation in Seoul
Source: Front Vet Sci. 2026 Sep 23;13:1887137. doi: 10.3389/fvets.2026.1887137 (PMC13643904; doi:10.3389/fvets.2026.1887137)
Supplement: Supplementary file 1 [file Data_Sheet_1.docx]

Supplementary Material

# 1 Supplementary Figures and Tables

## 1.1 Supplementary Figures

**Supplementary Figure 1.** Representative field recording sheet used for individual identification and data collection.

**Supplementary Figure 2.** Representative photographic examples matched with the morphological characteristic chart (Supplementary Figure 1) for individual identification of free-roaming cats.

## 1.2 Supplementary Tables

**Supplementary Table** **1.** **Site-level summary of free-roaming cat population metrics (2015–2025)**

| Year | Site_ID | Land_use | Area_km^2^ | Mean_cats | cat density | Neutered cats | Kitten |
| --- | --- | --- | --- | --- | --- | --- | --- |
| 2015 | S01 | Residential | 0.0703 | 32 | 455.5 | 5.0 | 14.0 |
| 2015 | S02 | Residential | 0.1038 | 28 | 269.9 | 11.0 | 9.0 |
| 2015 | S03 | Residential | 0.1042 | 22 | 211.2 | 1.0 | 9.0 |
| 2015 | S04 | Residential | 0.0772 | 42 | 544.4 | 2.0 | 5.0 |
| 2015 | S05 | Residential | 0.0749 | 26 | 347.2 | 6.0 | 10.0 |
| 2015 | S06 | Residential | 0.0798 | 32 | 400.9 | 0.0 | 16.0 |
| 2015 | S07 | Residential | 0.0678 | 23 | 339.2 | 3.0 | 6.0 |
| 2015 | S10 | Residential | 0.0989 | 44 | 445.1 | 2.0 | 22.0 |
| 2015 | S13 | Commercial | 0.0841 | 42 | 499.2 | 4.0 | 21.0 |
| 2017 | S01 | Residential | 0.0703 | 7 | 99.6 | 0.0 | 3.0 |
| 2017 | S02 | Residential | 0.1038 | 16 | 154.2 | 1.0 | 7.0 |
| 2017 | S03 | Residential | 0.1042 | 12 | 115.2 | 1.0 | 3.0 |
| 2017 | S04 | Residential | 0.0772 | 6 | 77.8 | 2.0 | 0.0 |
| 2017 | S05 | Residential | 0.0749 | 39 | 514.1 | 15.0 | 14.0 |
| 2017 | S06 | Residential | 0.0798 | 30 | 369.5 | 0.0 | 6.0 |
| 2017 | S07 | Residential | 0.0678 | 15 | 221.2 | 10.0 | 1.0 |
| 2017 | S08 | Residential | 0.0963 | 28 | 290.7 | 5.0 | 6.0 |
| 2017 | S09 | Residential | 0.0997 | 7 | 70.2 | 0.0 | 2.0 |
| 2017 | S10 | Residential | 0.0989 | 35 | 354.0 | 0.0 | 21.0 |
| 2017 | S11 | Residential | 0.0717 | 28 | 390.3 | 8.0 | 7.0 |
| 2017 | S12 | Commercial | 0.0989 | 12 | 121.4 | 0.0 | 4.0 |
| 2017 | S13 | Commercial | 0.0841 | 14 | 166.4 | 3.0 | 5.0 |
| 2017 | S14 | Industrial | 0.0974 | 36 | 364.6 | 1.0 | 21.0 |
| 2017 | S15 | Industrial | 0.0936 | 33 | 352.5 | 11.0 | 14.0 |
| 2017 | S16 | Green | 0.0830 | 15 | 180.6 | 0.0 | 14.0 |
| 2017 | S17 | Green | 0.0910 | 2 | 22.0 | 0.0 | 0.0 |
| 2019 | S01 | Residential | 0.0703 | 27 | 384.3 | 6.0 | 10.0 |
| 2019 | S02 | Residential | 0.1038 | 23 | 221.7 | 3.0 | 4.0 |
| 2019 | S03 | Residential | 0.1042 | 12 | 115.2 | 2.0 | 5.0 |
| 2019 | S04 | Residential | 0.0772 | 14 | 181.5 | 0.0 | 2.0 |
| 2019 | S05 | Residential | 0.0749 | 15 | 200.3 | 12.0 | 1.0 |
| 2019 | S06 | Residential | 0.0798 | 23 | 288.1 | 0.0 | 6.0 |
| 2019 | S07 | Residential | 0.0678 | 22 | 324.5 | 14.0 | 2.0 |
| 2019 | S08 | Residential | 0.0963 | 13 | 135.0 | 5.0 | 3.0 |
| 2019 | S09 | Residential | 0.0997 | 28 | 280.8 | 5.0 | 17.0 |
| 2019 | S10 | Residential | 0.0989 | 17 | 172.0 | 3.0 | 1.0 |
| 2019 | S11 | Residential | 0.0717 | 43 | 592.4 | 1.0 | 19.0 |
| 2019 | S12 | Commercial | 0.0989 | 13 | 131.5 | 3.0 | 4.0 |
| 2019 | S13 | Commercial | 0.0841 | 27 | 315.0 | 4.0 | 18.0 |
| 2019 | S14 | Industrial | 0.0974 | 16 | 164.3 | 8.0 | 0.0 |
| 2019 | S15 | Industrial | 0.0936 | 13 | 133.5 | 3.0 | 6.0 |
| 2019 | S16 | Green | 0.0830 | 6 | 66.2 | 1.0 | 2.0 |
| 2019 | S17 | Green | 0.0910 | 2 | 22.0 | 1.0 | 0.0 |
| 2021 | S01 | Residential | 0.0920 | 26 | 282.6 | 25.0 | 1.0 |
| 2021 | S02 | Residential | 0.1038 | 4 | 38.6 | 2.0 | 0.0 |
| 2021 | S03 | Residential | 0.1042 | 5 | 48.0 | 1.0 | 2.0 |
| 2021 | S04 | Residential | 0.0772 | 14 | 181.5 | 7.0 | 2.0 |
| 2021 | S05 | Residential | 0.0749 | 17 | 227.0 | 11.0 | 0.0 |
| 2021 | S06 | Residential | 0.0798 | 13 | 162.8 | 4.0 | 0.0 |
| 2021 | S07 | Residential | 0.0678 | 18 | 265.5 | 6.0 | 6.0 |
| 2021 | S08 | Residential | 0.0963 | 29 | 301.1 | 8.0 | 5.0 |
| 2021 | S09 | Residential | 0.0997 | 27 | 265.8 | 17.0 | 0.0 |
| 2021 | S10 | Residential | 0.0989 | 19 | 192.2 | 6.0 | 7.0 |
| 2021 | S11 | Residential | 0.0850 | 30 | 352.9 | 21.0 | 3.0 |
| 2021 | S12 | Commercial | 0.0989 | 2 | 20.2 | 1.0 | 0.0 |
| 2021 | S13 | Commercial | 0.0841 | 21 | 249.6 | 2.0 | 6.0 |
| 2021 | S14 | Industrial | 0.0974 | 14 | 143.8 | 8.0 | 2.0 |
| 2021 | S15 | Industrial | 0.0936 | 13 | 138.8 | 4.0 | 1.0 |
| 2021 | S16 | Green | 0.0830 | 19 | 228.8 | 5.0 | 3.0 |
| 2021 | S17 | Green | 0.0910 | 0 | 0.0 | 0.0 | 0.0 |
| 2023 | S01 | Residential | 0.0920 | 24 | 260.9 | 22.0 | 0.0 |
| 2023 | S02 | Residential | 0.1038 | 14 | 134.9 | 10.0 | 1.0 |
| 2023 | S03 | Residential | 0.1042 | 12 | 115.2 | 1.0 | 4.0 |
| 2023 | S04 | Residential | 0.0772 | 13 | 168.5 | 10.0 | 0.0 |
| 2023 | S05 | Residential | 0.0749 | 23 | 307.1 | 21.0 | 0.0 |
| 2023 | S06 | Residential | 0.0798 | 20 | 250.5 | 8.0 | 3.0 |
| 2023 | S07 | Residential | 0.0678 | 11 | 162.2 | 7.0 | 0.0 |
| 2023 | S08 | Residential | 0.0963 | 19 | 197.3 | 11.0 | 0.0 |
| 2023 | S09 | Residential | 0.0997 | 22 | 215.6 | 13.0 | 2.0 |
| 2023 | S10 | Residential | 0.0989 | 6 | 60.7 | 6.0 | 0.0 |
| 2023 | S11 | Residential | 0.0850 | 19 | 223.5 | 17.0 | 0.0 |
| 2023 | S12 | Commercial | 0.0989 | 6 | 60.7 | 4.0 | 0.0 |
| 2023 | S13 | Commercial | 0.0841 | 49 | 576.5 | 31.0 | 1.0 |
| 2023 | S14 | Industrial | 0.0974 | 10 | 102.7 | 5.0 | 2.0 |
| 2023 | S15 | Industrial | 0.0936 | 16 | 170.9 | 11.0 | 1.0 |
| 2023 | S16 | Green | 0.0830 | 18 | 210.7 | 11.0 | 1.0 |
| 2023 | S17 | Green | 0.0910 | 1 | 11.0 | 1.0 | 0.0 |
| 2025 | S01 | Residential | 0.0920 | 23 | 250.0 | 22.0 | 0.0 |
| 2025 | S02 | Residential | 0.1038 | 15 | 144.6 | 5.0 | 2.0 |
| 2025 | S03 | Residential | 0.1042 | 9 | 86.4 | 3.0 | 1.0 |
| 2025 | S04 | Residential | 0.0772 | 14 | 181.5 | 12.0 | 0.0 |
| 2025 | S05 | Residential | 0.0749 | 17 | 227.0 | 14.0 | 0.0 |
| 2025 | S06 | Residential | 0.0798 | 20 | 250.5 | 8.0 | 3.0 |
| 2025 | S07 | Residential | 0.0678 | 25 | 368.7 | 10.0 | 5.0 |
| 2025 | S08 | Residential | 0.0963 | 9 | 88.2 | 5.0 | 0.0 |
| 2025 | S09 | Residential | 0.0997 | 23 | 230.7 | 14.0 | 5.0 |
| 2025 | S10 | Residential | 0.0989 | 8 | 80.9 | 5.0 | 0.0 |
| 2025 | S11 | Residential | 0.0850 | 18 | 211.8 | 13.0 | 0.0 |
| 2025 | S12 | Commercial | 0.0989 | 10 | 101.1 | 2.0 | 5.0 |
| 2025 | S13 | Commercial | 0.0841 | 35 | 416.0 | 26.0 | 3.0 |
| 2025 | S14 | Industrial | 0.0974 | 6 | 61.6 | 6.0 | 0.0 |
| 2025 | S15 | Industrial | 0.0936 | 20 | 213.6 | 13.0 | 0.0 |
| 2025 | S16 | Green | 0.0830 | 5 | 60.2 | 2.0 | 0.0 |
| 2025 | S17 | Green | 0.0910 | 5 | 49.5 | 3.0 | 0.0 |

Survey boundaries at S01 and S11 were modestly adjusted from 2021 to include immediately adjacent areas in response to redevelopment. Area values are presented to four decimal places, whereas density calculations were based on the unrounded area values.

**Supplementary Table 2. Temporal changes in pooled cat density, site-level counts, neutering rate, and kitten proportion**

| **Year** | **Survey area (km²)** | **Total mean cats** | **Pooled density (cats/km²)** | **Mean cats/site ± SD** | **Neutering rate (%) ± SD** | **Kitten proportion (%) ± SD** | **Total count range (min–max)** | **Pooled density range (min–max, cats/km²)** |
| --- | --- | --- | --- | --- | --- | --- | --- | --- |
| 2017 | 1.49 | 334 | 223.5 | 19.6 ± 11.9 | 15.2 ± 19.4 | 33.8 ± 23.3 | 331–336 | 221.8-225.1 |
| 2019 | 1.49 | 312 | 209.0 | 18.4 ± 9.6 | 26.6 ± 22.6 | 27.6 ± 20.7 | 300–324 | 201.0-217.1 |
| 2021 | 1.53 | 271 | 177.1 | 15.9 ± 9.2 | 44.5 ± 22.3 | 13.9 ± 14.0 | 270–271 | 176.5–177.1 |
| 2023 | 1.53 | 282 | 184.3 | 16.6 ± 10.5 | 68.4 ± 23.2 | 5.8 ± 9.3 | 275–287 | 179.7–187.6 |
| 2025 | 1.53 | 261 | 170.9 | 15.4 ± 8.4 | 60.6 ± 23.3 | 8.2 ± 13.3 | 260–262 | 169.9–171.2 |

Total mean cats represent the sum of site-level mean counts across the 17 survey sites and are presented as rounded whole numbers. Total count ranges were calculated by summing site-level minimum and maximum counts, and pooled density ranges were obtained by dividing those totals by the corresponding year-specific total surveyed area. Pooled densities were calculated using unrounded site-level mean counts and unrounded site-specific area values and do not represent the arithmetic mean of site-specific densities. Mean cats per site, neutering rate, and kitten proportion are presented as mean ± SD across survey sites.

**Supplementary Table 3. Observed population indicators by land-use category and survey year (2017-2025)**

| **Year** | **Land-use category** | **Total mean count** | **Pooled density (cats/km²)** | **Mean site-level neutering rate (% ± SD)** | **Mean site-level kitten proportion (% ± SD)** |
| --- | --- | --- | --- | --- | --- |
| 2017 | Residential | 222 | 235.1 | 18.2 ± 21.6 | 28.2 ± 17.1 |
|  | Non-residential | 112 | 203.5 | 9.6 ± 14.3 | 44.0 ± 30.9 |
| 2019 | Residential | 237 | 250.4 | 24.7 ± 26.1 | 26.1 ± 17.9 |
|  | Non-residential | 76 | 137.8 | 30.1 ± 15.8 | 30.5 ± 26.8 |
| 2021 | Residential | 202 | 205.7 | 48.9 ± 23.1 | 14.1 ± 15.8 |
|  | Non-residential | 69 | 125.9 | 34.8 ± 19.1 | 13.3 ± 10.6 |
| 2023 | Residential | 183 | 186.3 | 68.3 ± 26.8 | 5.9 ± 10.5 |
|  | Non-residential | 99 | 180.6 | 68.7 ± 16.7 | 5.7 ± 7.5 |
| 2025 | Residential | 181 | 184.3 | 60.4 ± 21.9 | 7.4 ± 8.9 |
|  | Non-residential | 81 | 146.9 | 61.0 ± 27.8 | 9.8 ± 20.0 |

The same 17 monitoring sites, comprising 11 residential and six non-residential sites, were surveyed in all years. Non-residential sites included commercial, semi-industrial, and green areas. Cat densities were calculated using the corresponding year-specific survey areas reported in Supplementary Table 1, based on unrounded area values. Neutering rates and kitten proportions are presented as mean ± SD of site-level percentages. Values are descriptive; model-based comparisons are presented in Table 2 and Supplementary Table 7.

**Supplementary Table 4. Conditional single-visit detection probability estimated from repeated within-year encounter histories**

| Year | Encounter histories | Recorded visits per cat | Single-visit p | 95% CI | Standardized five-visit non-detection, % |
| --- | --- | --- | --- | --- | --- |
| Pooled | 1,478 | 5-7 | 0.299 | 0.287-0.312 | 16.9 |
| 2017 | 336 | 5 | 0.262 | 0.235–0.288 | 22.0 |
| 2019 | 324 | 5–6 | 0.277 | 0.250–0.304 | 19.8 |
| 2021 | 271 | 5–7 | 0.287 | 0.259–0.315 | 18.4 |
| 2023 | 285 | 5–6 | 0.336 | 0.308–0.365 | 12.9 |
| 2025 | 262 | 5 | 0.344 | 0.314–0.374 | 12.1 |

p, estimated single-visit detection probability; CI, confidence interval. Standardized probability of non-detection over five visits was calculated as (1 − p)^5 for all years to facilitate comparison. For individuals with six or seven recorded survey opportunities, the actual probability of remaining undetected across all available visits would be lower.

**Supplementary Table 5. Descriptive and adjusted analyses of apparent retention across consecutive survey intervals** **in 17 fixed survey sites**

**Panel A. Descriptive apparent retention across consecutive survey intervals**

| **Survey interval** | **Baseline neutering-status group** | **Baseline N** | **Re-identified n** | **Not re-identified n** | **Apparent retention, n/N (%)** |
| --- | --- | --- | --- | --- | --- |
| 2019–2021 | All cats | 324 | 37 | 287 | 37/324 (11.4) |
|  | Baseline-neutered | 71 | 20 | 51 | 20/71 (28.2) |
|  | Baseline-non-neutered | 229 | 17 | 212 | 17/229 (7.4) |
|  | Unknown baseline status | 24 | 0 | 24 | 0/24 (0.0) |
| 2021–2023 | All cats | 271 | 82 | 189 | 82/271 (30.3) |
|  | Baseline-neutered | 127 | 58 | 69 | 58/127 (45.7) |
|  | Baseline-non-neutered | 143 | 24 | 119 | 24/143 (16.8) |
|  | Unknown baseline status | 1 | 0 | 1 | 0/1 (0.0) |
| 2023–2025 | All cats | 285 | 83 | 202 | 83/285 (29.1) |
|  | Baseline-neutered | 189 | 68 | 121 | 68/189 (36.0) |
|  | Baseline-non-neutered | 92 | 15 | 77 | 15/92 (16.3) |
|  | Unknown baseline status | 4 | 0 | 4 | 0/4 (0.0) |

Apparent retention was defined as the proportion of individually identifiable cats observed at the earlier survey that were re-identified at the same survey site during the subsequent survey. Neutering status was assigned according to the earlier survey of each interval (baseline status). Cats that were non-neutered at baseline but were re-identified as ear-tipped at the subsequent survey remained classified in the baseline non-neutered group. The “All cats” category includes observations with unknown baseline neutering status, whereas status-specific rows include only cats with known baseline status. Apparent retention is an observation-based measure and should not be interpreted as a direct measure of survival, movement, or true site fidelity.

**Panel B. Binomial logistic mixed-effects model of apparent retention**

| **Predictor** | **Comparison** | **OR** | **95% CI** | **p-value** |
| --- | --- | --- | --- | --- |
| Baseline neutering status | Neutered vs. non-neutered | 3.062 | 2.070–4.528 | <0.001 |
| Baseline age class | Kitten vs. adult | 0.340 | 0.156–0.738 | 0.006 |
| Land-use category | Non-residential vs. residential | 0.938 | 0.581–1.516 | 0.795 |
| Survey interval | 2021–2023 vs. 2019–2021 | 2.202 | 1.387–3.494 | <0.001 |
| Survey interval | 2023–2025 vs. 2019–2021 | 1.611 | 0.998–2.601 | 0.051 |

Apparent retention at the subsequent survey was modeled as a binary outcome using a binomial logistic mixed-effects model. The model included baseline neutering status, baseline age class, land-use category, and survey interval as fixed effects and survey site as a random intercept. The reference categories were baseline non-neutered status, adult age class, residential land use, and the 2019–2021 interval. The adjusted model included 851 observations with known baseline neutering status; 14 observations with unknown baseline neutering status were excluded because all were classified as not retained, resulting in complete separation. OR, odds ratio; CI, confidence interval.

**Supplementary Table 6. Model-predicted neutering probabilities by survey year**

| **Survey year** | **Predicted neutering probability** | **SE** | **95% CI** |
| --- | --- | --- | --- |
| 2017 | 14.0% | 0.0291 | 9.16%–20.7% |
| 2019 | 18.9% | 0.0360 | 12.84%–27.0% |
| 2021 | 42.7% | 0.0555 | 32.28%–53.7% |
| 2023 | 66.5% | 0.0507 | 55.97%–75.6% |
| 2025 | 60.3% | 0.0544 | 49.31%–70.3% |

Predicted probabilities were estimated from the binomial GLMM for neutering status and back-transformed from the logit scale to the probability scale. Estimates were averaged over land-use categories.

**Supplementary Table 7. Negative binomial mixed-effects model evaluating the interaction between survey year and land-use category for adult cat counts**

| **Predictor** | **Estimate** | **SE** | **z** | **p-value** | **IRR** | **95% CI** |
| --- | --- | --- | --- | --- | --- | --- |
| Year, non-residential slope | -0.0325 | 0.0308 | -1.055 | 0.2915 | 0.9680 | 0.9112–1.0283 |
| Residential land use | 0.4094 | 0.2937 | 1.394 | 0.1634 | 1.5058 | 0.8468–2.6777 |
| Year × residential land use | -0.0006 | 0.0379 | -0.015 | 0.9883 | 0.9994 | 0.9280–1.0764 |

Estimates were obtained from a negative binomial GLMM for adult cat counts. The interaction term tested whether the temporal trend differed between residential and non-residential sites. The non-significant interaction indicates no evidence of different temporal slopes by land-use type.
